# New-Onset Musculoskeletal Manifestations in Hidradenitis Suppurativa: A Large-Scale Incidence Analysis

**DOI:** 10.7759/cureus.108229

**Published:** 2026-05-04

**Authors:** Isha Wilson, Gianna Haskin, Nima Karodeh, Destyni Hubbard, Jared Hall, Syed Fahad Gillani, Mekdem Bisrat, Anand Deonarine, Nicholas Azinge, Miriam B Michael

**Affiliations:** 1 Internal Medicine, Howard University College of Medicine, Washington D.C., USA; 2 Medicine and Surgery, King Edward Medical University, Lahore, PAK; 3 Internal Medicine, Howard University Hospital, Washington D.C., USA; 4 Internal Medicine, Howard University, Washington D.C., USA; 5 Internal Medicine, University of Maryland, Baltimore, USA

**Keywords:** cohort study, enthesopathy, hidradenitis suppurativa, inflammatory arthritis, joint pain, musculoskeletal manifestations, synovitis

## Abstract

Background

Hidradenitis suppurativa (HS) is a chronic inflammatory skin disease traditionally considered cutaneous in nature. Emerging data suggest HS may also have systemic inflammatory effects, including musculoskeletal involvement, yet large-scale studies evaluating incident joint disease following HS diagnosis are limited.

Objective

To quantify the 12-month incidence of new-onset musculoskeletal manifestations, including synovitis, enthesopathy, and small joint pain, among patients with newly diagnosed HS.

Methods

We conducted a retrospective cohort study using the TriNetX US Collaborative Network, analyzing electronic health records from 71 healthcare organizations. Patients aged 18-40 years with newly diagnosed HS and no prior history of joint disease were included. Outcomes assessed within 12 months of HS diagnosis included synovitis, tenosynovitis, enthesopathy, and small joint pain. Kaplan-Meier survival and frequency analyses were performed.

Results

Among 125,181 patients with HS, 1.2% developed synovitis or enthesopathy, and 2.3% developed small joint pain within one year of diagnosis. Kaplan-Meier survival probabilities at 12 months were 98.5% for synovitis/enthesopathy-free survival and 97.2% for small joint pain-free survival. The median number of outcome instances per patient was 1, with mean instances of 2.14 and 2.24, respectively.

Conclusion

This large-scale cohort study demonstrates that HS is associated with an early risk of musculoskeletal involvement, reinforcing its classification as a systemic inflammatory condition. Clinicians should monitor for joint-related symptoms in HS patients, particularly within the first year of diagnosis, to enable timely intervention and prevent progression.

## Introduction

Hidradenitis suppurativa (HS) is a chronic, relapsing inflammatory skin disorder characterized by painful nodules, abscesses, and sinus tracts, often in intertriginous areas. The predominant affected patient population is young adults, with the prevalence estimations varying by region. HS is associated with profound impacts on quality of life and healthcare utilization [1,2]. 

Traditionally accepted as a disease limited to the skin, HS is increasingly recognized to have systemic inflammatory effects. Patients with HS often exhibit comorbid conditions, such as obesity, dyslipidemia, type 2 diabetes, and metabolic syndrome, indicating a burden beyond the standard dermatologic symptoms [3-5]. 

Emerging evidence also links HS with rheumatologic and musculoskeletal manifestations. Recent studies have reported an increased prevalence of psoriatic arthritis, Spondyloarthritis, and other joint diseases in HS populations [6-9]. Cohort analyses suggest that the risk of new onset inflammatory arthritis, such as ankylosing spondylitis, psoriatic arthritis, and rheumatoid arthritis, is significantly greater among patients after HS diagnosis is made compared with matched non-HS controls [10, 11]. 

Despite the rise in data accumulation, prior work has limitations. Many studies have a cross-sectional design, small sample sizes, or insufficient temporal data to assess the incidence of joint disease within defined windows after HS onset. Therefore, there is a need for larger-scale analyses with sufficiently defined inclusion criteria to better quantify the risk of musculoskeletal involvement after HS diagnosis [12, 13]. 

To address these gaps, we conducted a large retrospective cohort study using the TriNetX US Collaborative Network to quantify the 12-month risk of musculoskeletal manifestations, including enthesopathy, synovitis, and small joint pain, among patients with newly diagnosed HS.

## Materials and methods

Study design and data source

We conducted a retrospective cohort study using the TriNetX US Collaborative Network, which includes de-identified electronic health records from 71 healthcare organizations across the United States. Data available through TriNetX comprises diagnoses, procedures, medications, laboratory values, and demographic information.

Cohort selection

Patients were eligible if they were between 18 and 40 years of age and had a diagnosis of HS [HS; International Classification Of Diseases (ICD)-10-Clinical Modification (CM): L73.2]. To ensure inclusion of incident cases without prior joint disease, patients were excluded if they had a history of rheumatoid arthritis (M06), polyosteoarthritis (M15, M15.9), osteoarthritis of the knee (M17), osteoarthritis of the hip (M16), gout (M10), systemic lupus erythematosus (M32), juvenile rheumatoid arthritis (M08.00), ankylosing spondylitis (M45), or psoriatic arthritis (L40.50). The final cohort included 125,739 patients who met these criteria.

Follow-up periods and outcomes

The index date was defined as the first recorded diagnosis of HS (HS; ICD-10-CM: L73.2) that met eligibility criteria. Outcomes were assessed beginning one day after the index date and continued through 365 days of follow-up. Patients whose index event occurred 20 or more years before cohort entry were excluded (n=558). The primary outcomes of interest were musculoskeletal manifestations. Specifically, synovitis and tenosynovitis (M65), enthesopathies of the lower limb excluding the foot (M76), and other enthesopathies (M77) were grouped as the “synovitis and enthesopathy” outcome. In addition, small joint pain outcomes included pain in the wrist (M25.53), pain in joints of the hand (M25.54), and pain in the ankle and joints of the foot (M25.57). All outcomes were evaluated only if they occurred within the defined 365-day observation window following the index event.

Statistical analysis

We used the TriNetX Analyze Outcomes module to evaluate the study outcomes. Risk analyses were performed to estimate the proportion of patients who developed each outcome within the 12-month follow-up period. Survival analyses were conducted using the Kaplan-Meier method to estimate time to outcome, with censoring at the last recorded encounter. From these analyses, we reported the median survival time and the 12-month survival probability. In addition, several instances of analysis were performed to calculate the frequency distribution of outcome events, reporting the mean, median, and standard deviation across patients. All analyses were conducted using the default TriNetX platform settings. Sensitivity analyses for excluding patients with outcomes prior to the observation window were considered; however, for this report, patients with prior outcomes were retained.

## Results

Cohort characteristics

A total of 125,739 patients with hidradenitis suppurativa (HS) were identified in the TriNetX US Collaborative Network after applying inclusion and exclusion criteria. Of these, 558 patients were excluded because their index event occurred ≥20 years before cohort entry, leaving 125,181 patients for analysis. Please see the age and sex characteristics of HS patients.

**Table 1:** Age and Sex Characteristics of HS patients in the cohort.

Variable	Patients	% of Cohort	Mean ± SD	Median	Min	Max
Age						
Current Age	125,181	100%	30.4 ± 6.12	31	18	40
Age at Index	125,181	100%	25 ± 6.54	25	0	40
Sex						
Female	99,064	79.14%	—	—	—	—
Male	23,771	18.99%	—	—	—	—
Unknown	2,346	1.87%	—	—	—	—

Synovitis and enthesopathy outcomes

Risk Analysis

Within one year of HS diagnosis, 1,542 patients (1.2%) developed synovitis, tenosynovitis, or enthesopathy.

**Table 2:** Incidence and event frequency of synovitis, tenosynovitis, and enthesopathy within 12 months of hidradenitis suppurativa diagnosis. Data are presented as numbers and percentages of affected patients (N, %) and as mean ± standard deviation (SD) and median number of events among those with outcomes. A total of 1,542 of 125,181 patients (1.2%) developed outcomes. Among affected patients, the mean number of instances was 2.14±2.74, with a median of 1. Statistical significance was defined as p<0.05. ICD: International Classification Of Diseases.

Variable	Value
ICD 10 Codes	M65 (Synovities and Tenosynovities), M76 (Enthospoathies of lower limb, excluding foot), M77 (Other enterosopathies)
Patients in Cohort	125,181
Patients with outcome	1,542
Risk(%)	1.23
Mean Instance	2.14
Standard Deviation (SD)	2.74
Median	1
12-month survival probability	98.48%
Median survival (days)	Not Reached (Survival didn’t drop below 50%)

Survival Analysis

**Figure 1:**
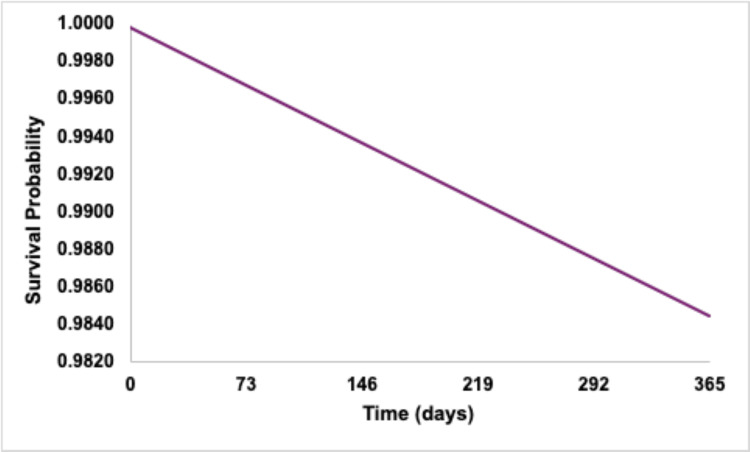
Kaplan–Meier curve demonstrating event-free survival for synovitis, tenosynovitis, and enthesopathy within 12 months following diagnosis of hidradenitis suppurativa. The 12-month event-free survival probability was 98.5%. Event rates are derived from a total cohort of N=125,181 patients, with outcomes occurring in 1,542 patients (1.2%). Tick marks represent censored observations. Statistical significance was defined as p<0.05.

Kaplan-Meier analysis demonstrated a 12-month survival probability of 98.5% without outcome occurrence (Figure 1). Median survival time could not be estimated because fewer than 50% of patients experienced the outcome during follow-up.

Small joint pain outcomes

Risk Analysis

A total of 2,831 patients (2.3%) developed small joint pain (wrist, hand, ankle, or foot) within one year of HS diagnosis (Table 2)

**Table 3:** Incidence and frequency of small joint pain within 12 months of hidradenitis suppurativa diagnosis. Data are presented as numbers and percentages (N, %) of affected patients and as mean ± standard deviation (SD) and median number of occurrences among those with outcomes. A total of 2,831 of 125,181 patients (2.3%) developed small joint pain. Among affected patients, the mean number of instances was 2.24±2.95, with a median of 1. Statistical significance was defined as p<0.05. ICD: International Classification Of Diseases.

Variable	Value
ICD 10 Codes	M25.35 (Pain in wrist), M25.54 (Pain in joints of hand), M25.54 (Pain in ankle and joints of foot)
Patients in Cohort	125,181
Patients with outcome	2,831
Risk (%)	2.26
Mean Instance	2.24
Standard Deviation (SD)	2.95
Median	1
12-month survival probability	97.21%
Median survival (days)	Not reached (Survival didn’t drop below 50%)

Survival Analysis

**Figure 2:**
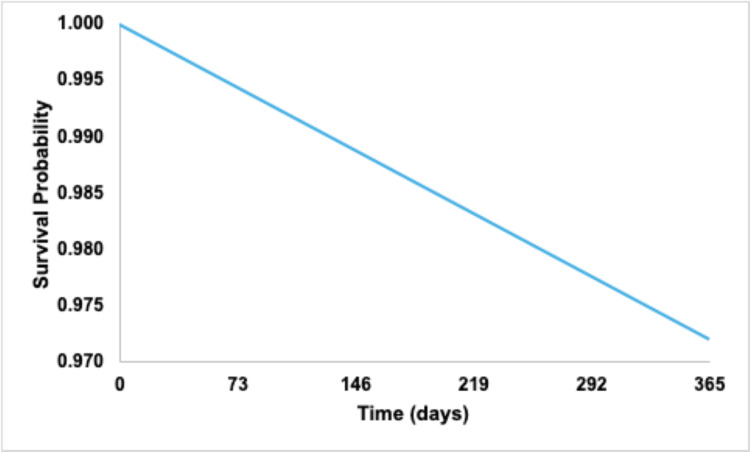
Kaplan–Meier survival curve for small joint pain.

Kaplan-Meier analysis demonstrated a 12-month survival probability of 97.2% without outcome occurrence (Figure 2). Median survival time was not reached within the 12-month follow-up window.

## Discussion

Our study, leveraging a large, multicenter cohort of 125,181 patients with newly diagnosed HS, provides robust real-world evidence on the early risk of musculoskeletal manifestations in this population. Within the first year after HS diagnosis, 1.2% of patients developed synovitis or enthesopathy, and 2.3% developed small joint pain. Kaplan-Meier survival probabilities at 12 months were 98.5% for synovitis/enthesopathy-free survival and 97.2% for small joint pain-free survival. Among those affected, the median number of outcome instances per patient was 1, with mean instances of 2.14 for synovitis/enthesopathy and 2.24 for small joint pain. These findings indicate that while joint involvement is measurable early in the disease course, it remains relatively uncommon in the first year [1-4].

Our results align with prior studies reporting an elevated risk of rheumatologic comorbidities in HS populations. Schneeweiss et al. and others have demonstrated that patients with HS are at increased risk of developing inflammatory arthritis, including spondyloarthritis and rheumatoid arthritis, compared to the general population [10]. Richette et al. found that musculoskeletal symptoms often develop several years after HS onset, with a mean interval of 3.6 years between skin and joint symptom onset, and a crude prevalence of spondyloarthritis of 3.7% in HS cohorts [6]. Meta-analyses confirm a two to three-fold increased risk of inflammatory arthritis in HS patients, with risk factors including younger age, male sex, and severe disease [7, 8]. Our study adds to this literature by quantifying the early, incident risk in a large, diverse U.S. population, and by applying strict exclusion criteria to minimize confounding from preexisting autoimmune or joint disease. It complements broader word characterizing HS as a systemic inflammatory disease with heterogeneous comorbidity burden [14, 15]. Studies examining immune-mediated mechanisms and comorbidity clustering further support the systemic nature of HS [5].

Importantly, our data show that early joint involvement in HS is present but occurs at low rates, reinforcing the concept that HS is primarily a cutaneous and follicular disease in its initial stages [1, 2, 9]. However, longitudinal studies and case-control analyses consistently demonstrate that the risk of musculoskeletal manifestations increases with disease duration and severity [11-13]. For example, Garbayo-Salmons et al. reported that HS patients with arthritis have higher rates of immune-mediated comorbidities, greater impairment in quality of life, and increased use of biologic therapies compared to those with HS alone [16]. MRI-based studies have revealed a high prevalence of back pain and axial spondyloarthropathy in patients with moderate to severe HS, with up to 39% showing signs of active spondyloarthritis [13]. The risk of osteoarthritis is also elevated in HS, with a 1.37-fold higher risk compared to non-HS controls over a one to five-year follow-up [12, 17].

Clinical implications 

Clinically, our results highlight the importance of vigilance for joint-related symptoms in HS populations, even in the early disease course. Dermatologists and rheumatologists should routinely assess for musculoskeletal complaints and maintain a low threshold for referral, as early recognition may allow for timely intervention and prevention of chronic disability [5, 15]. Our findings support an integrated, multidisciplinary approach to HS care, extending beyond skin symptoms to encompass comorbidity screening and coordination with rheumatology and primary care. As the risk of joint involvement increases with disease progression, ongoing surveillance and early intervention protocols are warranted, particularly for patients with risk factors such as younger age, male sex, and severe HS [1, 5, 6, 9].

Limitations 

Several limitations of our study should be acknowledged. The reliance on ICD-10 codes introduces the potential for misclassification, as coding practices may vary across institutions. The TriNetX platform does not provide granular clinical data on HS severity, treatment exposures, or lifestyle risk factors, which may mediate musculoskeletal outcomes. Our one-year follow-up period captures only early incident cases and does not reflect longer-term risk trajectories. Extended longitudinal studies are needed to clarify whether risk increases over time or varies according to HS severity and treatment response. Despite these limitations, our study's strengths include its large sample size, multicenter U.S. coverage, and standardized analytic approach, which improve generalizability and provide robust estimates of short-term musculoskeletal incidence after HS diagnosis.

## Conclusions

Our study demonstrates that early joint involvement in HS is present but uncommon, with measurable incidence of synovitis, enthesopathy, and small joint pain within the first year of diagnosis. These results provide further evidence that HS is a systemic inflammatory disease and underscore the need for integrated care and ongoing surveillance to address both cutaneous and musculoskeletal health needs. By quantifying early risk and contextualizing it within the broader disease trajectory, our study informs clinical practice and supports the development of targeted screening and intervention strategies for HS patients.
